# 800 Firefighter Burn Center Visits and the Benefit to Providers, Patients, and Firefighter Volunteers

**DOI:** 10.1093/jbcr/iraf019.331

**Published:** 2025-04-01

**Authors:** Mickey Randahl, Caitlin Orton, Gretchen Carrougher, Tam Pham

**Affiliations:** Washington State Council of Firefighter Burn Foundation; University of Washington; University of Washington; University of Washington

## Abstract

**Introduction:**

Firefighter Burn Center (FBC) visits are an opportunity for firefighters to meet with inpatients, providers and ambulatory patients and families. At one burn center, 1-1.5 hour monthly FBC visits have occurred for several years. During these visits, firefighters engage with burn center personnel, patients and families, providing visitation, comradery, and gifting (e.g., goodie bags, toys, and refreshments). The aim of this project was to evaluate the perceived value of FBC visits and the partnership that has been created between the burn center and local fire departments.

**Methods:**

Two surveys, guided by the National Association of County and City Health Official’s Partnership Evaluation Guide, were developed to understand the perceived value of FBC visits. Using an online URL link or QR code, surveys were sent to firefighters who visited a burn center and providers who experienced a visit in the past year. During a one-week evaluation period, respondents were asked to respond to multiple questions using a 5-point Likert evaluation scale. Surveys were designed to assess the value of the program with open-ended questions intended to capture perspectives and the ability for the program to build community partnerships. Respondent comments were analyzed using thematic analysis.

**Results:**

23 firefighters and 28 providers responded. Most firefighters strongly agreed that visits were meaningful personally (91.3%). Providers responded with 85.7% strongly agreed/agreed to the question, ‘The firefighter burn center visits have been beneficial for myself’. Providers also reported strong agreement (82.1%) that firefighter visits have been beneficial to patients, noting that visits have helped improve patients’ mood and engagement in their care. Thematic analysis revealed 3 common themes: Community relations - visits provide opportunities to build and maintain community connections; Job resiliency – interactions reinforced the “why” respondents chose their professions and continue to do their work; Need for program expansion – to include other shifts and other local hospitals. Firefighters also noted that visits provided an opportunity to learn about burn injury recovery.

**Conclusions:**

This evaluation determined the existing FBC visit model has a positive impact on the local burn community with facilitation of building community relations between burn providers, patients, survivors and the firefighter community.

**Applicability of Research to Practice:**

Other fire departments and burn centers can implement similar programs to build community, and support patients during recovery and aftercare.

**Funding for the Study:**

N/A

